# Broussoflavonol B from *Broussonetia kazinoki* Siebold Exerts Anti-Pancreatic Cancer Activity through Downregulating FoxM1

**DOI:** 10.3390/molecules25102328

**Published:** 2020-05-16

**Authors:** Ji Hye Jeong, Jae-Ha Ryu

**Affiliations:** Research Institute of Pharmaceutical Sciences and College of Pharmacy, Sookmyung Women’s University, Seoul 04310, Korea; jjh4415@naver.com

**Keywords:** pancreatic cancer, Broussoflavonol B, *Broussonetia kazinoki* Siebold, FoxM1

## Abstract

Pancreatic cancer has a high mortality rate due to poor rates of early diagnosis. One tumor suppressor gene in particular, p53, is frequently mutated in pancreatic cancer, and mutations in p53 can inactivate normal wild type p53 activity and increase expression of transcription factor forkhead box M1 (FoxM1). Overexpression of FoxM1 accelerates cellular proliferation and cancer progression. Therefore, inhibition of FoxM1 represents a therapeutic strategy for treating pancreatic cancer. Broussoflavonol B (BF-B), isolated from the stem bark of *Broussonetia kazinoki* Siebold has previously been shown to inhibit the growth of breast cancer cells. This study aimed to investigate whether BF-B exhibits anti-pancreatic cancer activity and if so, identify the underlying mechanism. BF-B reduced cell proliferation, induced cell cycle arrest, and inhibited cell migration and invasion of human pancreatic cancer PANC-1 cells (p53 mutated). Interestingly, BF-B down-regulated FoxM1 expression at both the mRNA and protein level. It also suppressed the expression of FoxM1 downstream target genes, such as cyclin D1, cyclin B1, and survivin. Cell cycle analysis showed that BF-B induced the arrest of G0/G1 phase. BF-B reduced the phosphorylation of extracellular signal-regulated kinase ½ (ERK½) and expression of ERK½ downstream effector c-Myc, which regulates cell proliferation. Furthermore, BF-B inhibited cell migration and invasion, which are downstream functional properties of FoxM1. These results suggested that BF-B could repress pancreatic cancer cell proliferation by inactivation of the ERK/c-Myc/FoxM1 signaling pathway. Broussoflavonol B from *Broussonetia kazinoki* Siebold may represent a novel chemo-therapeutic agent for pancreatic cancer.

## 1. Introduction

Pancreatic cancer is one of the most lethal human malignancies with a five-year survival rate of around 9% [1]. Because of the absence of characteristic symptoms, early diagnosis is rare and metastasis rates are high, resulting in poor survival. In the last few decades, Gemcitabine and 5-Fluorouracil (5-FU) have been the most commonly used chemotherapeutic agents for pancreatic cancer, but overall the therapeutic efficacy is not satisfactory [2]. Therefore, an urgent need exists to develop new drugs for treating pancreatic cancer.

Approximately 70% of pancreatic cancers have p53 gene mutations [3,4] and most p53 mutations directly disrupt the protein’s DNA-binding activity [5]. Inactivation of wild-type p53 by loss or mutation of the p53 gene leads to chemotherapy resistance, reduces metabolic regulation, and increases metastasis [6]. In addition, expression of FoxM1 increases after p53 mutation or deletion [7,8]. FoxM1 is an oncogenic transcription factor that plays important roles in the initiation, progression, metastasis, and drug resistance of a variety of human tumors, including pancreatic cancer [9,10]. FoxM1 is a critical cell cycle regulator of both the G1/S and G2/M transitions and functions by regulating transcription of cell cycle genes [11]. Previous studies showed that FoxM1 is highly expressed in multiple human cancers such as glioblastoma [12], breast cancer [13], and colorectal cancer [14]. Therefore, effective inhibition of FoxM1 could contribute to reduced tumorigenesis and cancer progression.

**Figure 1:**
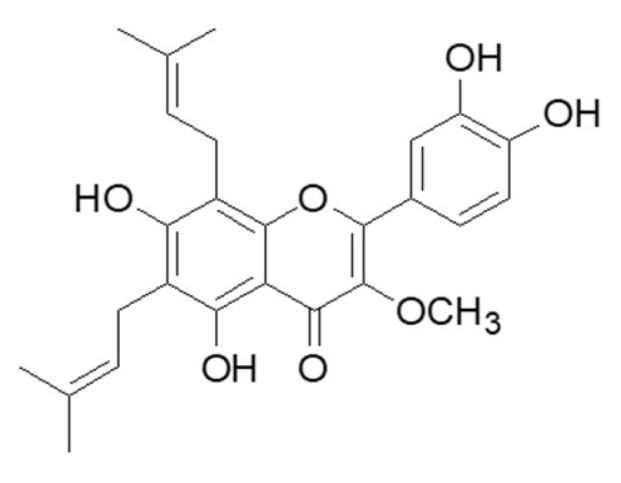
The chemical structure of 5,7,3′,4′-tetrahydroxy-3-methoxy-6,8-diprenylflavone (BF-B).

*Broussonetia kazinoki* Siebold (paper mulberry, Moraceae) is distributed throughout the world including in East Asia and the Pacific Islands. Since ancient times, it has been used to treat various ailments and its properties have been thought to strengthen the liver and kidneys, nourish the eyes, and treat edema [15]. The bioactive substances in this plant have been reported to have anti-inflammatory [16], anticancer [17], and anti-melanogenic activity [18]. Broussoflavonol B (5,7,3′,4′-tetrahydroxy-3-methoxy-6,8-diprenylflavone (BF-B)) (Figure 1) isolated from stem bark of *Broussonstia kazinoki* Siebold, was reported to exert anti-inflammatory [19], anti-breast cancer [20,21], and cholinesterase inhibitory activities [22]. In the present study, anti-pancreatic cancer activity of BF-B was demonstrated through down-regulating FoxM1 that is responsible for tumorigenesis and invasion of p53 mutated cancers.

## 2. Results

### 2.1. BF-B Reduces Viability of Human Pancreatic Cancer PANC-1 Cells

**Figure 2:**
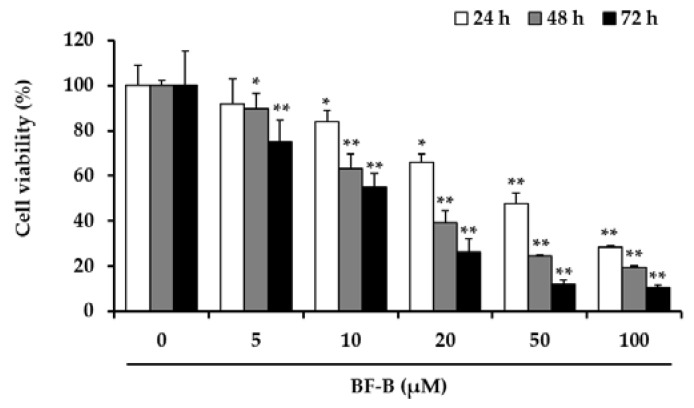
Effect of BF-B on growth of PANC-1 cells. PANC-1 cells were treated with BF-B at 0, 5, 10, 20, 50, and 100 µM, for 24, 48, or 72 h, and cell viability was assessed by MTT assay. All data are expressed as means ± SD of three experiments and each experiment included triplicate repeats. * *p* < 0.01 and ** *p* < 0.001 as compared to vehicle control.

Several prenylated flavonoids from medicinal plants were reported to exhibit cytotoxic activity on different cancer cell types [23,24], and a metabolite of flavonoids was recently suggested as the regulator of cyclin dependent kinase [25]. To determine the effect of BF-B on cell viability of pancreatic cancer cell, the cytotoxic effects were measured by 3-(4,5-dimethylthiazol-2-yl)2,5-diphenyl-tetrazolium bromide (MTT) assay. As shown in Figure 2, BF-B significantly inhibited the viability of PANC-1 cells in a dose-dependent manner. The 50% inhibitory concentration of cell viability (IC_50_) of BF-B for 1, 2, and 3 days of treatment were 43, 20.4, and 11.2 μM, respectively. These results show that BF-B reduced proliferation of pancreatic cancer cells.

### 2.2. BF-B Inhibits FoxM1 Expression

**Figure 3:**
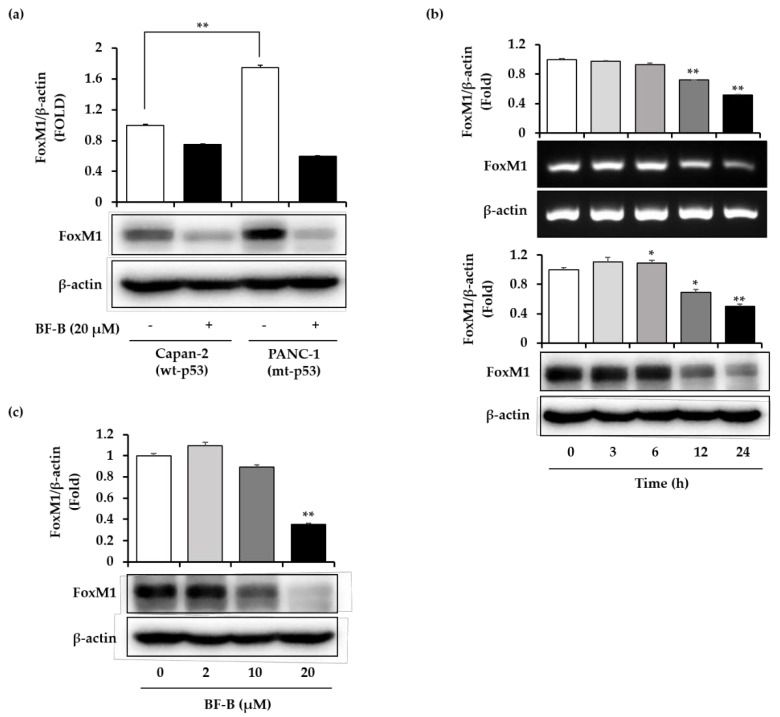
Inhibition of FoxM1 expression by BF-B. (**a**) Human pancreatic cancer cells (Capan-2 and PANC-1) were treated with 20 µM BF-B for 24 h. The level of FoxM1 was determined by Western blotting. ** *p* < 0.001 (**b**) PANC-1 cells were treated with 20 µM BF-B for the indicated times. FoxM1 mRNA was detected by real-time RT-PCR and the FoxM1 protein was detected by Western blotting. * *p* < 0.01 and ** *p* < 0.001 as compared to control. (**c**) PANC-1 cells were treated with the indicated concentrations of BF-B for 24 h. FoxM1 protein was detected by Western blotting. The images are representative of three independent experiments with similar results. All data are expressed as means ± SD of three experiments and each experiment included triplicate repeats. ** *p* < 0.001 as compared to control.

In pancreatic cancer, FoxM1 was reported to play an important role in cell growth and proliferation [9], and to show particularly high expression in p53 mutated tumors [26]. Accordingly, the expression pattern of FoxM1 was compared between a p53 wild-type (Capan-2) and a p53 mutated (PANC-1) pancreatic cancer cell line (Figure 3a). The expression level of FoxM1 in PANC-1 cells was 1.7 times higher than in Capan-2 cells.

To investigate the inhibitory effect of BF-B on FoxM1 expression, PANC-1 cells were exposed to BF-B. BF-B time-dependently decreased the expression of FoxM1 at both mRNA and protein levels (Figure 3b). BF-B treatment for 24 h significantly suppressed the expression of FoxM1 protein in a dose-dependent manner (Figure 3c). These results indicate that BF-B inhibits the expression of FoxM1 and this activity might be responsible for inhibiting the cell growth of pancreatic cancer cells.

### 2.3. BF-B Induces G0/G1 Arrest through Down-Regulating FoxM1 Target Genes

**Figure 4:**
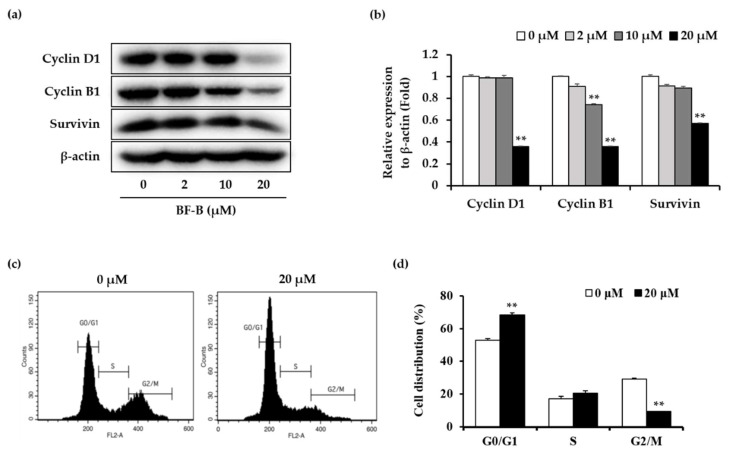
Effect of BF-B on FoxM1 target gene expression and cell cycle phase distribution in PANC-1 cells. (**a**) PANC-1 cells were treated with the indicated concentrations of BF-B for 24 h, and protein levels of cyclin D1, cyclin B1, and survivin were detected by Western blotting and (**b**) each protein levels were quantified using Image J software. ** *p* < 0.001 as compared to control. (**c**) PANC-1 cells were treated with 20 µM of BF-B for 24 h. Cell cycle distribution was assessed by flow cytometric analysis. (**d**) The cell population in each stage of the cell cycle was determined using BD CellQuest Pro software. All data are expressed as means ± SD of three experiments and each experiment included triplicate repeats. ** *p* < 0.001 as compared to control of each group.

FoxM1 is known to regulate transcription of cell proliferation associated proteins, such as cyclin B, cyclin D, cyclin A, Cdk2, p21, p27, Cdc25, and survivin [27,28,29,30]. To further investigate the regulatory effect of BF-B on FoxM1, the expression of downstream FoxM1 target genes was evaluated. BF-B dose-dependently repressed the expression of cyclin D1, cyclin B1, and survivin (Figure 4a,b).

To determine the effects on cell cycle distribution, PANC-1 cells were stained with propidium iodide (PI). BF-B significantly increased the population of cells in G0/G1 phase of the cell cycle from 53% (control) to 68.3% after treatment with 20 μM for 24 h (Figure 4c,d). These data reveal that BF-B induces G0/G1 phase arrest and suppresses the proliferation of PANC-1 cells through down-regulating FoxM1 target genes.

### 2.4. BF-B Inhibits Cell Migration and Invasion of Pancreatic Cancer Cells

**Figure 5:**
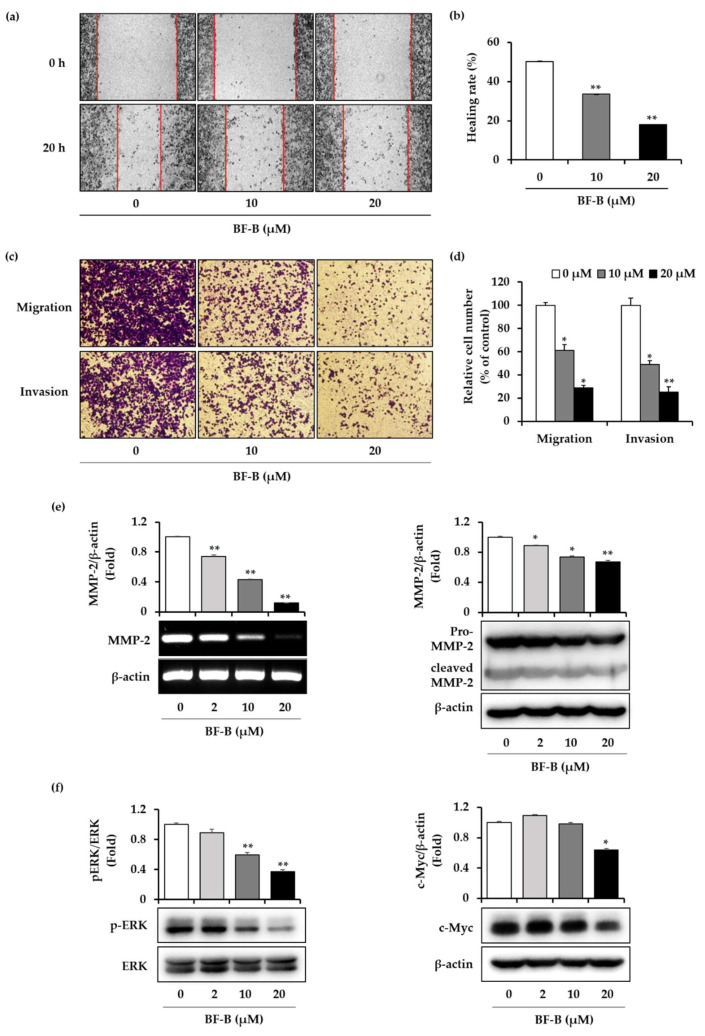
Effect of BF-B on migration and invasion of PANC-1 cells. (**a**,**b**) PANC-1 cells were treated with BF-B at the indicated concentrations for 20 h, and then subjected to wound healing migration assay. The wound distance was measured at 0 and 20 h to calculate healing rate as explained in methods. ** *p* < 0.001 as compared to control. (**c**,**d**) Cells were treated with BF-B at the indicated concentrations for 20 h, and then subjected to transwell migration and invasion assay. Transmigrated and invaded cells were counted from the five photographed fields in each group. * *p* < 0.01 and ** *p* < 0.001 as compared to control. (**e**,**f**) PANC-1 cells were treated with BF-B at the indicated concentrations for 20 h. The level of MMP-2 mRNA was evaluated by real-time PCR and level of MMP-2, phospho ERK½, ERK½, and c-Myc were detected by Western blotting. Images are representative of three independent experiments with similar results. * *p* < 0.01 and ** *p* < 0.001 as compared to control.

FoxM1 regulates cell migration and invasion of pancreatic cancer cells [27]. To assess whether BF-B influences the migration and invasion of PANC-1 cells, wound healing and invasion assays was performed. In terms of wound healing, treatment with BF-B, 10 and 20 µM for 20 h, reduced the healing rate by 33.5% and 18%, respectively, compared with 0 h control group (Figure 5a,b). Consistent with the results of the wound healing assay, BF-B reduced the cell migration and invasion in assays using noncoated and matrigel-coated chambers. BF-B treatment (10 and 20 µM, 20 h) reduced cell migration by 61.2%and 28.9%, and reduced the number of cells invading into the lower chambers by 49% and 25.1%, respectively (Figure 5c,d).

MMP-2 has been reported to play critical roles in cancer invasion and metastasis [31]. To identify the effect of BF-B on MMP-2 expression, PANC-1 cells treated with various concentrations of (0, 2, 10, and 20 μM) of BF-B for 20 h. As shown in Figure 5e, BF-B dose-dependently decreased the expression of MMP-2 at both mRNA and protein levels.

To investigate further the underlying mechanisms behind BF-B-mediated anti-invasive and anti-migratory activity in PANC-1 cells, the expression of ERK was examined by Western blot analysis. ERK pathway is an important regulator of cancer development and progression [32]. As shown in Figure 5f, BF-B suppressed the phosphorylation of ERK as well as the expression of its target gene, c-Myc in PANC-1 cells, in a dose-dependent manner. These results demonstrate that BF-B inhibits migration and invasion of pancreatic cancer cells through downregulating ERK signaling pathways.

## 3. Discussion

Broussoflavonol B purified from *Broussonetia kazinoki* Siebold was reported to be cytotoxic against breast cancer cells. In estrogen receptor (ER)-positive breast cancer MCF7 cells, BF-B inhibited cell growth through down-regulation of ER-α36 expression. ER-α36, a variant of ER-α, was known to correlate with carcinogenesis, aggressiveness, and therapeutic resistance of breast cancer [33]. BF-B also inhibited growth of ER-negative breast cancer MDA-MB-231 cells through downregulation of ER-α36 and induction of the G0/G1 and G2/M phase arrest [21]. However, there are no reports on any anti-pancreatic cancer activity of BF-B. Therefore, the anticancer effect of BF-B on human pancreatic cancer cells was investigated, and the underlying mechanism was explored, specifically focused on FoxM1 inhibition.

The effect of BF-B on the proliferation of PANC-1 and Capan-2 cells was compared using MTT assay. BF-B inhibited proliferation of both cells in a dose and time dependent manners, though the effect on PANC-1 cells was slightly higher than Capan-2 cells (Figure S1a). p53, one of the most frequently mutated genes in cancer, is mutated in about 70% of pancreatic cancers [34]. When mutated, cell cycle regulation is disrupted resulting in abnormal cell proliferation [35]. As a proliferation-associated transcription factor, forkhead box M1 (FoxM1) has an essential role in cell cycle progression. Expression of FoxM1 is up-regulated in p53 mutated cancers [26], and conversely can be repressed by p53 activation [8]. Loss of FoxM1 expression in pancreatic cancer suppresses cancer progression and metastasis in vitro and in vivo [36]. The proliferation rate of PANC-1 cells was about four-times higher than Capan-2 cells (Figure S1b). The doubling time of PANC-1 and Capan-2 cells was reported as 52 h [37] and 96 h [38], respectively. Capan-2 has wild-type p53, while PANC-1 has mutant p53 that leads to the high expression of FoxM1. The expression level of FoxM1 in PANC-1 cells was 1.7-times higher than that of Capan-2 cells (Figure 3a), which could be responsible for the higher proliferation rate of PANC-1 cells. FoxM1 plays important roles in tumorigenesis, proliferation, and therapeutic resistance, especially in p53-mutated cancers like PANC-1 cells. Accordingly, PANC-1 cells were used to study the anti-pancreatic cancer activity of BF-B, as an inhibitor of FoxM1 expression. Meanwhile, both PANC-1 and Capan-2 cells have K-ras mutation in common [39]. So, K-ras might be another target of BF-B for its anti- cancer activity. BF-B did not show any cytotoxicity on normal colon cells (CCD-18Co) (Figure S2).

FoxM1 has been shown to be down-regulated by several phytochemicals, including genestein [28], diarylheptanoids [40] and Z-ajoene [41]. These observations suggest that inhibition of FoxM1 is a potentially useful strategy for cancer therapy especially in p53 mutated cancers. Accordingly, we have tried to discover FoxM1 inhibitor from medicinal plants as anti-cancer agents.

The results revealed that BF-B significantly attenuated cancer cell growth and viability. Furthermore, BF-B suppressed the expression of FoxM1 and its target genes such as cyclin D1, cyclin B1 and survivin, resulting in cell cycle arrest in G0/G1 phase of PANC-1 cells.

Cell migration and invasion are important processes in tumor metastasis and FoxM1 is known to control these processes [9]. In pancreatic cancer cells, down-regulation of FoxM1 reduced the expression of MMP-2 and MMP-9, resulting in the inhibition of migration and invasion [27]. FoxM1 directly regulates the expression of MMP-2 at the transcriptional level to promote glioma progression [42]. As shown in Figure 5, BF-B not only reduced the migration and invasion of PANC-1 cells, but also significantly inhibited the expression of MMP-2 at both mRNA and protein levels.

The ERK signaling pathway is a prototypic mitogen-activated protein kinase (MAPK) signaling cascade that is critical in cell motility, although it is classically known as an important regulator of cell proliferation, differentiation, and survival. ERK signaling is activated in response to growth factors and the extracellular matrix (ECM) to increase proliferation of tumor cells [43]. Mutant p53 promotes a sustained EGF-induced ERK½ activation, thereby facilitating cell proliferation and tumorigenesis [44]. Elevated FoxM1 expression is triggered by the constitutive activation of ERK, which leads to enhanced cell motility in ovarian cancer [45]. BF-B significantly suppressed phosphorylated-ERK and its target gene, c-Myc which is critical in controlling cell proliferation. c-Myc is known to regulate the expression of FoxM1 by binding to its promoter region in prostate cancer [46].

In summary, broussoflavonol B from *Broussonetia kazinoki* Siebold is an active compound with anti-pancreatic cancer effect. It suppressed the expression of FoxM1 and its target genes to induce G0/G1 phase arrest in p53 mutant PANC-1 cells. BF-B also inhibited cell migration and invasion through reducing ERK activity and MMP-2 expression in PANC-1 cells. Thus, these results suggest that broussoflavonol B may possess potential as a novel chemo-preventative agent against pancreatic cancer.

## 4. Materials and Methods

### 4.1. Extraction and Isolation

The dried stem bark of *Broussonetia kazinoki* Siebold (2 kg) was extracted with 70% ethanol with reflux for 2 h and evaporated to generate extracts. The concentrated extracts (90 g) were suspended in water and partitioned with ethyl acetate. The ethyl acetate soluble fraction (20 g) was chromatographed over silica gel column with *n*-hexane/ethyl acetate gradient elution (50:1→1:1, *v*/*v*) to obtain 15 fractions. Subsequently, fraction 9 (503 mg) was subjected to column chromatography on silica gel eluting with methylene chloride/methanol (100:1→1:5, *v*/*v*) to give 10 fractions. Subfraction F9-3 (170 mg) was further separated on a RP-C18 column with a gradient elution of acetonitrile (40%→100%) to yield broussoflavonol B (34 mg). The structure of broussoflavonol B was identified by spectroscopic analyses of NMR (Varian INOVA 400 MHz, Varian Inc., Palo Alto, CA, USA), Mass (Agilent 6530 Q-TOF mass spectrometer, Agilent, Santa Clara, CA, USA) and IR (FT/IR-430, Jasco Inc., Tokyo, Japan) [47].

### 4.2. Cell Culture

PANC-1 cells were purchased from the American Type Culture Collection (ATCC; Manassas, VA, USA) and cultured in Dulbecco’s Modified Eagle Medium (DMEM; Gibco, Gran Island, NY, USA) supplemented with 10% heat-inactivated fetal bovine serum (FBS; TCB, Tulare, CA, USA), 100 U/mL penicillin and 10 μg/mL streptomycin. Capan-2 cells were kindly provided by Prof. Yong-Yeon Cho, The Catholic University of Korea (Bucheon-si, Korea) and cultured in RMPI1640 (Corning Inc., Corning, NY, USA) supplemented with 10% heat-inactivated fetal bovine serum (FBS; TCB, Tulare, CA, USA), 100 U/mL penicillin and 10 μg/mL streptomycin. All cells were maintained in a humidified atmosphere with 5% CO_2_ at 37 °C.

### 4.3. MTT Assay

Cells were seeded into 96 well plates at 2 × 10^3^ cells/well, allowed to adhere overnight and incubated with various concentrations of BF-B for 1–3 days. After treatment, MTT solution (0.5 mg/mL) was added and incubated for 2 h at 37 °C. Then, 100 μL DMSO was added to each well, and the plate was put on a shaker for 5 min. Absorbance were measured at 540 nm with a microplate reader (Molecular Devices, Sunnyvale, CA, USA) [48].

### 4.4. RT-PCR

Total RNA from cultured cells was extracted using TRIzol™ Reagent (Invitrogen, Carlsbad, CA, USA) in accordance with the manufacturer’s instructions. First-stand cDNA was synthesized using Labopass^TM^ cDNA synthesis kit (Cosmogenetech, Seoul, Korea) and amplified using a PCR thermal cycler (GeneAmp PCR System 2700, Applied Biosystems, Foster City, CA, USA). The amplified DNA was separated on 2% agarose gels and stained with ethidium bromide. Primers used in this study were as follows: FoxM1: forward, 5′—ATGGCAAAT TTTTCGCTCC—3′, and reverse, 5′—ATGTCACCAGAAATTCCCAGTT—3′; MMP-2: forward, 5′—CACTTTCCTGGGCAACAAAT—3′, and reverse, 5′—TGATGTCATCCTGGGACAGA—3′; β-actin: forward, 5′—AAGGGACTTCCTGTAACAACG—3′, and reverse, 5′—AGGATGCAGAAGGAGATCACT—3′.

### 4.5. Western Blot

Total cell lysates were prepared using RIPA buffer (50 mM Tris-HCl, pH 7.6, 150 mM NaCl, 1% Triton X-100, 1% sodium deoxycholate and protease inhibitor cocktail). Clear protein extracts were obtained by centrifugation at 15,000 rpm at 4 °C for 30 min. The protein concentration was determined by bicinchoninic acid (BCA™)-based protein assay (Thermo Scientific, Rockford, IL, USA). Equal amounts of lysates (20 μg protein/lane) were subjected to 8–12% SDS-PAGE and transferred onto polyvinylidene difluoride (PVDF) membrane (GE Healthcare, Chicago, IL, USA). PVDF membranes were blocked with 5% nonfat dry milk for 1 h at room temperature and incubated with primary antibodies against FoxM1 (A301–533A, Bethyl Laboratories, Montgomery, TX, USA), cyclin D1, cyclin B1, survivin, phosphorylated extracellular signal-regulated kinase (p-ERK), total ERK, c-Myc (#2922, #4138, #2808, #9101, #9102, #9402, Cell Signaling Technology, Danvers, MA, USA), MMP-2 (sc-10736, Santa Cruz Biotechnology, Santa Cruz, CA, USA), and β-actin (A2066, Sigma Aldrich, St. Louis, MO, USA) overnight at 4 °C. This was followed by incubation for 2 h at room temperature with horseradish peroxidase (HRP)-conjugated secondary antibody (1:10,000 dilution). β-actin was used to normalize protein loading. The membranes were detected using ECL detection system (GE Healthcare, Chicago, IL, USA) and analyzed by Image J software (NIH, Bethesda, MD, USA).

### 4.6. Cell Cycle Analysis

PANC-1 cells were treated with 20 μM BF-B for 24 h, harvested, washed with cold PBS, and fixed with 70% ethanol at 4 °C overnight. Cell pellets were resuspended in PBS and incubated with RNase A solution for 1 h at 37 °C. Propidium iodide (PI) was added to cells and incubated at 4 °C for 20 min. Cells (10,000) were analyzed using a FACS Calibur flow cytometer (BD Biosciences, San Jose, CA, USA). The results were analyzed with BD CellQues™ Pro software (version 6.0, BD Biosciences, San Jose, CA, USA).

### 4.7. Wound Healing Assay

PANC-1 cells were seeded in 6-well plates and incubated until 90% confluent. Wounds were created with a pipette tip and then cells were washed with PBS. Medium containing BF-B was added to the cells and incubated for 24 h. The scraped wound areas were observed and photographed under a microscope (Olympus, Tokyo, Japan) at 0 and 20 h. The wound distance was measured using Image-J software (NIH, Bethesda, MD, USA). The relative wound healing rate (%) were calculated according to the formula 1 − [D_20_]/[D_0_], with [D_20_] and [D_0_] being the gap distance at 20 and 0 h, respectively [49].

### 4.8. Transwell Migration and Invasion Assays

BD BioCoat^TM^ Matrigel^TM^ Invasion Chamber (BD Bioscience, San Jose, CA, USA) was used to study cell migration and invasion. The transwell chamber system was used directly for cell migration, while matrigel coated chambers (BD Biosciences, San Jose, CA, USA) were used for cell invasion. Cells treated with BF-B (50,000 in 200 μL of serum free medium) were added to each insert, while 750 μL of serum-containing medium was added to each lower chamber. Plates were incubated at 37 °C for 20 h with 5% CO_2_ to allow the cells to pass through the pores on the membrane. Afterward, remaining cell suspension was removed and the inserts were fixed in formaldehyde solution (3.7% in PBS) and stained with 0.1% crystal violet for 15 min. The stained cells were photographed and counted under a microscope (Olympus, Tokyo, Japan) at 100× magnification [50,51].

### 4.9. Statistical Analysis

The Student’s *t*-test was used to compare means between control and experimental groups. All experiments were performed three times. Results are expressed as the mean ± standard deviation (S.D.). *p* values < 0.01 were considered statistically significant.

## Supplementary Materials

The following are available online. Figure S1: Effects of BF-B on proliferation of Capanc-2 and PANC-1 human pancreatic cancer cells. (**a**) Cells were treated with BF-B at the indicated concentrations for 24 h and 72 h. (**b**) Cells were treated with BF B at the indicated concentrations for 72 h. Cell viability was determined by MTT assay. Values are presented as mean ± S.D. * *p* < 0.01, ** *p* < 0.001 as compared to the respective control of Capan-2 cells. Figure S2: Effects of BF-B on proliferation of CCD-18Co normal colon cells.

## References

[1] Siegel R.L., Miller K.D., Jemal A. (2019). Cancer statistics, 2019. CA A Cancer J. Clin..

[2] Yue Q., Gao G., Zou G., Yu H., Zheng X. (2017). Natural Products as Adjunctive Treatment for Pancreatic Cancer: Recent Trends and Advancements. BioMed Res. Int..

[3] Rozenblum E., Schutte M., Goggins M., Hahn S.A., Panzer S., Zahurak M., Goodman S.N., Sohn T.A., Hruban R.H., Yeo C.J. (1997). Tumor-suppressive pathways in pancreatic carcinoma. Cancer Res..

[4] Scarpa A., Capelli P., Mukai K., Zamboni G., Oda T., Iacono C., Hirohashi S. (1993). Pancreatic adenocarcinomas frequently show p53 gene mutations. Am. J. Pathol..

[5] Kato S., Han S.Y., Liu W., Otsuka K., Shibata H., Kanamaru R., Ishioka C. (2003). Understanding the function-structure and function-mutation relationships of p53 tumor suppressor protein by high-resolution missense mutation analysis. Proc. Natl. Acad. Sci. USA.

[6] Muller P.A., Vousden K.H. (2014). Mutant p53 in cancer: New functions and therapeutic opportunities. Cancer Cell.

[7] Pandit B., Halasi M., Gartel A.L. (2009). p53 negatively regulates expression of FoxM1. Cell Cycle.

[8] Barsotti A.M., Prives C. (2009). Pro-proliferative FoxM1 is a target of p53-mediated repression. Oncogene.

[9] Huang C., Du J., Xie K. (2014). FOXM1 and its oncogenic signaling in pancreatic cancer pathogenesis. Biochim. Biophys. Acta.

[10] Huang C., Zhang X., Jiang L., Zhang L., Xiang M., Ren H. (2019). FoxM1 Induced Paclitaxel Resistance via Activation of the FoxM1/PHB1/RAF-MEK-ERK Pathway and Enhancement of the ABCA2 Transporter. Mol. Ther. Oncolytics.

[11] Kalin T.V., Ustiyan V., Kalinichenko V.V. (2011). Multiple faces of FoxM1 transcription factor: Lessons from transgenic mouse models. Cell Cycle.

[12] Liu M., Dai B., Kang S., Ban K., Huang F., Lang F.F., Aldape K.D., Xie T., Pelloski C.E., Xie K. (2006). FoxM1B Is Overexpressed in Human Glioblastomas and Critically Regulates the Tumorigenicity of Glioma Cells. Cancer Res..

[13] Madureira P.A., Varshochi R., Constantinidou D., Francis R.E., Coombes R.C., Yao K., Lam E.W. (2006). The Forkhead Box M1 Protein Regulates the Transcription of the Estrogen Receptor α in Breast Cancer Cells. J. Biol. Chem..

[14] Chu X.Y., Zhu Z.M., Chen L.B., Wang J.H., Su Q.S., Yang J.R., Lin Y., Xue L.J., Liu X.B., Mo X.B. (2012). FOXM1 expression correlates with tumor invasion and a poor prognosis of colorectal cancer. Acta Histochem..

[15] Wang G.W., Huang B.K., Qin L.P. (2012). The genus Broussonetia: A review of its phytochemistry and pharmacology. Phytother. Res..

[16] Lee D.Y., Lee H.J., Ryu J.H. (2018). Prenylated Polyphenols from Broussonetia kazinoki as Inhibitors of Nitric Oxide Production. Molecules.

[17] Kim H.S., Lim J., Lee D.Y., Ryu J.H., Lim J.S. (2015). Kazinol C from Broussonetia kazinoki activates AMP-activated protein kinase to induce antitumorigenic effects in HT-29 colon cancer cells. Oncol. Rep..

[18] Lim J., Nam S., Jeong J.H., Kim M.J., Yang Y., Lee M.S., Lee H.G., Ryu J.H., Lim J.S. (2019). Kazinol U inhibits melanogenesis through the inhibition of tyrosinase-related proteins via AMP kinase activation. Br. J. Pharm..

[19] Ryu H.W., Park M.H., Kwon O., Kim D., Hwang J., Jo Y.H., Ahn K.S., Hwang B.Y., Oh S. (2019). Anti-inflammatory flavonoids from root bark of Broussonetia papyrifera in LPS-stimulated RAW264.7 cells. Bioorganic Chem..

[20] Guo F., Feng L., Huang C., Ding H., Zhang X., Wang Z., Li Y. (2013). Prenylflavone derivatives from Broussonetia papyrifera, inhibit the growth of breast cancer cells in vitro and in vivo. Phytochem. Lett..

[21] Guo M.X., Wang M.L., Deng H., Zhang X.T., Wang Z.Y. (2013). A novel anticancer agent Broussoflavonol B downregulates estrogen receptor (ER)-α36 expression and inhibits growth of ER-negative breast cancer MDA-MB-231 cells. Eur. J. Pharm..

[22] Ryu H.W., Curtis-Long M.J., Jung S., Jeong I.Y., Kim D.S., Kang K.Y., Park K.H. (2012). Anticholinesterase potential of flavonols from paper mulberry (Broussonetia papyrifera) and their kinetic studies. Food Chem..

[23] Wei S., Sun T., Du J., Zhang B., Xiang D., Li W. (2018). Xanthohumol, a prenylated flavonoid from Hops, exerts anticancer effects against gastric cancer in vitro. Oncol. Rep..

[24] Wang P., Zhang J., Xiong X., Yuan W., Qin S., Cao W., Dai L., Xie F., Li A., Liu Z. (2019). Icariin suppresses cell cycle transition and cell migration in ovarian cancer cells. Oncol. Rep..

[25] Sankaranarayanan R., Valiveti C.K., Kumar D.R., Van Slambrouck S., Kesharwani S.S., Seefeldt T., Scaria J., Tummala H., Bhat G.J. (2019). The Flavonoid Metabolite 2,4,6-Trihydroxybenzoic Acid Is a CDK Inhibitor and an Anti-Proliferative Agent: A Potential Role in Cancer Prevention. Cancers.

[26] Parikh N., Hilsenbeck S., Creighton C.J., Dayaram T., Shuck R., Shinbrot E., Xi L., Gibbs R.A., Wheeler D.A., Donehower L.A. (2014). Effects of TP53 mutational status on gene expression patterns across 10 human cancer types. J. Pathol..

[27] Wang Z., Banerjee S., Kong D., Li Y., Sarkar F.H. (2007). Down-regulation of Forkhead Box M1 transcription factor leads to the inhibition of invasion and angiogenesis of pancreatic cancer cells. Cancer Res..

[28] Wang Z., Ahmad A., Banerjee S., Azmi A., Kong D., Li Y., Sarkar F.H. (2010). FoxM1 is a novel target of a natural agent in pancreatic cancer. Pharm. Res..

[29] Wang I.C., Chen Y.J., Hughes D., Petrovic V., Major M.L., Park H.J., Tan Y., Ackerson T., Costa R.H. (2005). Forkhead box M1 regulates the transcriptional network of genes essential for mitotic progression and genes encoding the SCF (Skp2-Cks1) ubiquitin ligase. Mol. Cell. Biol..

[30] Radhakrishnan S.K., Bhat U.G., Hughes D.E., Wang I.C., Costa R.H., Gartel A.L. (2006). Identification of a chemical inhibitor of the oncogenic transcription factor forkhead box M1. Cancer Res..

[31] Dong W., Li H., Zhang Y., Yang H., Guo M., Li L., Liu T. (2011). Matrix metalloproteinase 2 promotes cell growth and invasion in colorectal cancer. Acta Biochim. Biophys. Sin..

[32] Guo Y.-J., Pan W., Liu S., Shen Z., Xu Y., Hu L. (2020). ERK/MAPK signalling pathway and tumorigenesis. Exp. Ther. Med..

[33] Su X., Xu X., Li G., Lin B., Cao J., Teng L. (2014). ER-α36: A novel biomarker and potential therapeutic target in breast cancer. Oncotargets Ther..

[34] Hwang R.F., Gordon E.M., Anderson W.F., Parekh D. (1998). Gene therapy for primary and metastatic pancreatic cancer with intraperitoneal retroviral vector bearing the wild-type p53 gene. Surgery.

[35] Kern S.E., Pietenpol J.A., Thiagalingam S., Seymour A., Kinzler K.W., Vogelstein B. (1992). Oncogenic forms of p53 inhibit p53-regulated gene expression. Science.

[36] Huang C., Qiu Z., Wang L., Peng Z., Jia Z., Logsdon C.D., Le X., Wei D., Huang S., Xie K. (2012). A novel FoxM1-caveolin signaling pathway promotes pancreatic cancer invasion and metastasis. Cancer Res..

[37] Lieber M., Mazzetta J., Nolson-Rees W., Kaplan M., Todaro G. (1975). Establishment of a continuous tumor-cell line (PANC-1) from a human carcinoma of the exocrine pancreas. Int. J. Cancer.

[38] Kyriazis A.A., Kyriazis A.P., Sternverg C.N., Sloane N.H., Loveless J.D. (1986). Morphological, Biological, Biochemical, and Karyotypic Characteristics of Human Pancreatic Ductal Adenocarcinoma Capan-2 in Tissue Culture and the Nude Mouse. Cancer Res..

[39] Brunner T.B., Cengel K.A., Hahn S.M., Wu J., Fraker D.L., Mckenna W.H., Bernhard E.J. (2005). Pancreatic Cancer Cell Radiation Survival and Prenyltransferase Inhibition: The Role of K-Ras. Cancer Res..

[40] Dong G.Z., Jeong J.H., Lee Y.I., Lee S.Y., Zhao H.Y., Jeon R., Lee H.J., Ryu J.H. (2017). Diarylheptanoids suppress proliferation of pancreatic cancer PANC-1 cells through modulating shh-Gli-FoxM1 pathway. Arch. Pharm. Res..

[41] Lee H.J., Jeong J.H., Ryu J.H. (2019). Anti-pancreatic cancer activity of Z-ajoene from garlic: An inhibitor of the Hedgehog/Gli/FoxM1 axis. J. Funct. Foods.

[42] Dai B., Kang S.H., Gong W., Liu M., Aldape K.D., Sawaya R., Huang S. (2007). Aberrant FoxM1B expression increases matrix metalloproteinase-2 transcription and enhances the invasion of glioma cells. Oncogene.

[43] Tanimura S., Takeda K. (2017). ERK signalling as a regulator of cell motility. J. Biochem..

[44] Wang W., Cheng B., Miao L., Mei Y., Varshochi R. (2013). Mutant p53-R273H gains new function in sustained activation of EGFR signaling via suppressing miR-27a expression. Cell Death Dis..

[45] Lok G.T., Chan D.W., Liu V.W., Hui W.W., Leung T.H., Yao K.M., Ngan H.Y. (2011). Aberrant activation of ERK/FOXM1 signaling cascade triggers the cell migration/invasion in ovarian cancer cells. Plos ONE.

[46] Pan H., Zhu Y., Wei W., Shao S., Rui X. (2018). Transcription factor FoxM1 is the downstream target of c-Myc and contributes to the development of prostate cancer. World J. Surg. Oncol..

[47] Matsumoto J., Fujimoto T., Takino C., Saitoh M., Hano Y., Fukai T., Nomura T. (1985). Components of *Broussonetia papyrifera* (L.) VENT. I. Structures of Two New Isoprenylated Flavonols and Two Chalcone Derivatives. Chem. Pharm. Bull..

[48] Khan M., Bajpai V.K., Kang S.C. (2017). Visual experiment MTT assay to evaluate the cytotoxicity potential of a drug. Bangladesh J. Pharmacol..

[49] Cao J., Wu Q., Zheng W., Li L., Mei W. (2017). Microwave-assisted synthesis of polypyridyl ruthenium(ii) complexes as potential tumor-targeting inhibitors against the migration and invasion of Hela cells through G2/M phase arrest. RSC Adv..

[50] Su J., Zhou X., Wang L., Yin X., Wang Z. (2016). Curcumin inhibits cell growth and invasion and induces apoptosis through down-regulation of Skp2 in pancreatic cancer cells. Am. J. Cancer Res..

[51] Gu X.D., Xu L.L., Zhao H., Gu J.Z., Xie X.H. (2017). Cantharidin suppressed breast cancer MDA-MB-231 cell growth and migration by inhibiting MAPK signaling pathway. Braz. J. Med. Biol. Res..

