# Supplementary material for: Broussoflavonol B from Broussonetia kazinoki Siebold Exerts Anti-Pancreatic Cancer Activity through Downregulating FoxM1
Source: Molecules. 2020 May 16;25(10):2328. doi: 10.3390/molecules25102328 (PMC7287790; doi:10.3390/molecules25102328)
Supplement: Supplementary file 1 [file molecules-25-02328-s001.pdf]

[Supplementary materials]

(a)

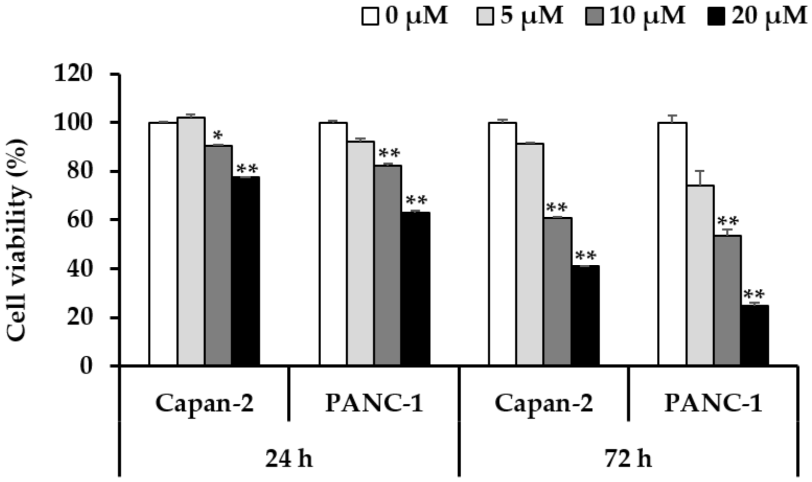

(b)

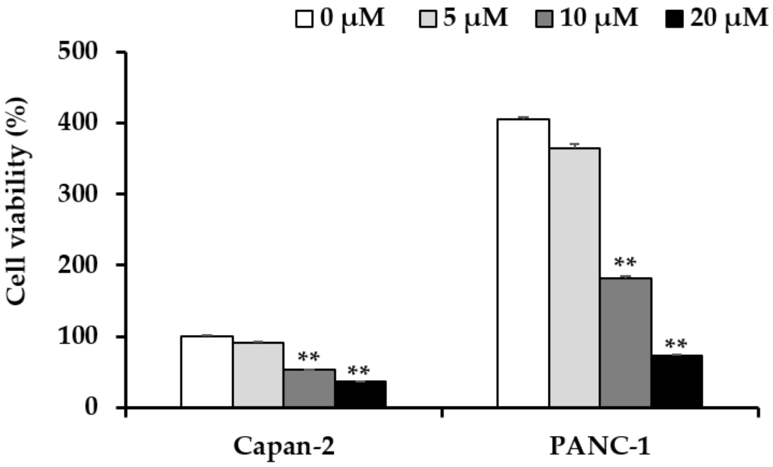

Figure S1. Effects of BF-B on proliferation of Capanc-2 and PANC-1 human pancreatic cancer cells. (a) Cells were treated with BF-B at the indicated concentrations for 24 h and 72 h. (b) Cells were treated with BF-B at the indicated concentrations for 72 h. Cell viability was determined by MTT assay. Values are presented as mean ± S.D. * $p < 0.01$, ** $p < 0.001$ as compared to the respective control of Capna-2 cells.

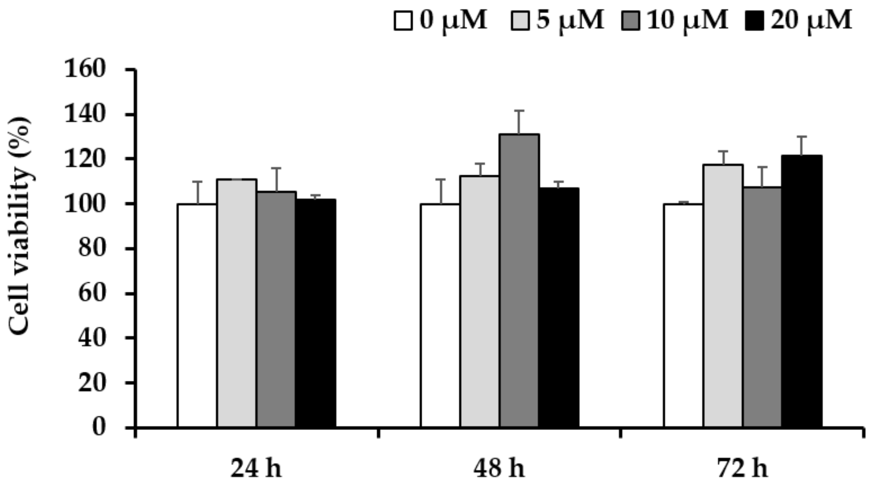

Figure S2. Effects of BF-B on proliferation of CCD-18Co normal colon cells. Cells were treated with BF-B at the indicated concentrations for 24 h, 48 h, and 72 h. Cell viability was determined by MTT assay. Values are presented as mean ± S.D.
